# Postoperative New-Onset Atrial Fibrillation following Noncardiac Operations: Prevalence, Complication, and Long-Term MACE

**DOI:** 10.1155/2020/8156786

**Published:** 2020-10-14

**Authors:** Ofir Koren, Rony Hakim, Asaf Israeli, Ehud Rozner, Yoav Turgeman

**Affiliations:** ^1^Heart Institute, Emek Medical Center, Afula, Israel; ^2^Bruce Rappaport Faculty of Medicine, Technion-Israel Institute of Technology, Haifa, Israel; ^3^Anesthesia Department, Emek Medical Center, Afula, Israel

## Abstract

**Background:**

Postoperative new-onset atrial fibrillation (POAF) is a common complication following cardiothoracic surgery, but little is known regarding its occurrence and outcome following noncardiothoracic surgery. This study was intended to examine the incidence of POAF in noncardiothoracic surgeries performed under general anesthesia and its effects on the length of hospitalization stay, short-term and long-term morbidity, and mortality. *Methodology*. We conducted a retrospective observational descriptive study. The study population consists of patients hospitalized in surgical wards from January 2014 to December 2017. Surgery was defined as noncardiac or thoracic procedure conducted under general anesthesia.

**Results:**

A total of 24,125 general anesthesia operations were performed at 7 surgical wards. About two-fifth of the operations (40%) were operated electively, and the rest underwent emergency surgery. The mean age was 63.78 ± 11.50, and more than half (56.9%) of the participants were female. The prevalence of POAF was 2.69 per 1000 adult patients (95% CI: 2.11–3.43) and vary significantly among wards. The highest prevalence was observed after hip fixation and laparotomy surgeries (54.9 and 26.7 per 1000 patients, respectively). The median length of hospitalization was significantly higher in POAF patients (21.0 vs. 4.8 days, *p* < 0.001). Patients who developed POAF had significantly higher mortality rates, both inhospital (200 vs. 7.56 deaths per 1000, *p*=0.001) and 1 year (261.5 vs. 33.3 per 1000, *p*=0.001, respectively). There was no significant association between outcome and treatment modalities such as rate or rhythm control and anticoagulant use.

**Conclusion:**

New-onset AF following noncardiac surgery is rare, yet poses significant clinical implications, both immediate and long-term. POAF is associated with a longer length of hospitalization and a significantly higher mortality rate, both in short- and long-term.

## 1. Introduction

Postoperative atrial fibrillation (POAF) is one of the most common complications in patients undergoing cardiac surgery. Its incidence is estimated to be about 30–60% of patients. The occurrence of atrial fibrillation after surgery results in longer hospitalization duration, higher morbidity and mortality, frequent recurrences, and long-term risk of stroke [1–3].

POAF has been extensively studied in the context of cardiac surgery (CS) [4–7], but little is known about its prevalence after noncardiac surgery (NCS). It is estimated that the prevalence is between 5 and 10% of patients and is dependent on many variables, such as age, cardiac dysfunction, and cardiovascular risk factors [8].

POAF is thought to occur as a result of adrenergic stimulation, systemic inflammation, or activation of the autonomous heart nerves during surgery and up to four days later, for various reasons. These reasons include pain, low blood pressure, infection, and bleeding. POAF appears to be associated with myocardial injury or hypertension even before surgery, possibly from hemodynamically alterations or electrolytic disorders [9].

The therapeutic and preventive approach is unclear. A rate control strategy does not appear to differ from rhythm control therapy as far as mortality is concerned [10, 11].

### 1.1. The Rationale of the Research

The purpose of this study was to examine the incidence of atrial fibrillation following noncardiothoracic surgeries. We also examined the risk factors for the appearance of POAF, its effect on length of hospitalization, inhospital morbidity and mortality, and abnormal cardiovascular events (MACE) in the short- and long-term follow-up.

## 2. Materials and Methods

This was a retrospective observational descriptive study, which was conducted in the heart institute of Emek Medical Center, Afula, Israel. This facility is a secondary, medium-sized medical center, with larger tertiary hospitals close by. As such, a majority of surgeries performed at this center are emergent. The study population consists of patients hospitalized in seven surgical wards (orthopedics, 2 surgical wards, obstetrics and gynecology, plastic surgery, otolaryngology (ENT, ear, nose, and throat), and urology) from January 1, 2014, to the end of December 2017. Surgery was arbitrarily defined by us as a surgical procedure performed under general anesthesia. Procedures performed with local, regional, or spinal anesthesia were excluded. Patients who met the inclusion criteria were divided based on the appearance of POAF. The development of POAF was observed in the 36-hour close monitoring following surgeries in our facility. We have collected data from the hospital's computer systems (“Orion, Ofek, and Chameleon”) in accordance with International Diagnostic Code 10-ICD.

Patients eligible for the study (Table 1, inclusion and exclusion criteria) were divided based on the primary endpoint which was defined as the appearance of new atrial fibrillation diagnosed during hospitalization. Secondary end points were major adverse cardiovascular events (MACE) defined as recurrent atrial fibrillation (AF), cerebrovascular accident (CVA), transient ischemic attack (TIA), and pulmonary embolism (PE) hospitalization due to congestive heart failure (CHF) and death at 30 days and at 1 year.

### 2.1. Ethics

The study was approved by the Ethics Committee of the hospital in accordance with the Helsinki Convention (no. EMC-0088-18). Informed consent was not required due to the confidentiality of patient data.

### 2.2. Sample Size

Considerations in calculating the sample size were based on several key points: rough assessment of annually conducted surgeries at our medical center and the incidence of POAF known from prior publication. We estimated an arbitrary minimal incidence rate of 2% for POAF and at least 15% group difference. Sample size calculation estimated a total of 4520 participants for 95% CI and 80% power. Preliminary data indicated a significantly lower incidence rate of POAF which led us to increase the sample size by elongating the study period.

### 2.3. Statistics

Categorical variables were presented using frequencies and percentages, and these continuous variables were presented using standard distribution indices (mean, standard deviation, median, etc.). Chi-squared test was used for analysis. Differences in the two group's demographic data were tested by *χ*^2^ test or Fisher's exact test appropriate for the categorical data and by student's *t*-test or Mann–Whitney *U* test in the case of nonparametric data for continuous data. The statistical processing will be carried out using SAS 9.4 software. Significance would be obtained if *p* < 0.05.

## 3. Results

**Figure 1:**
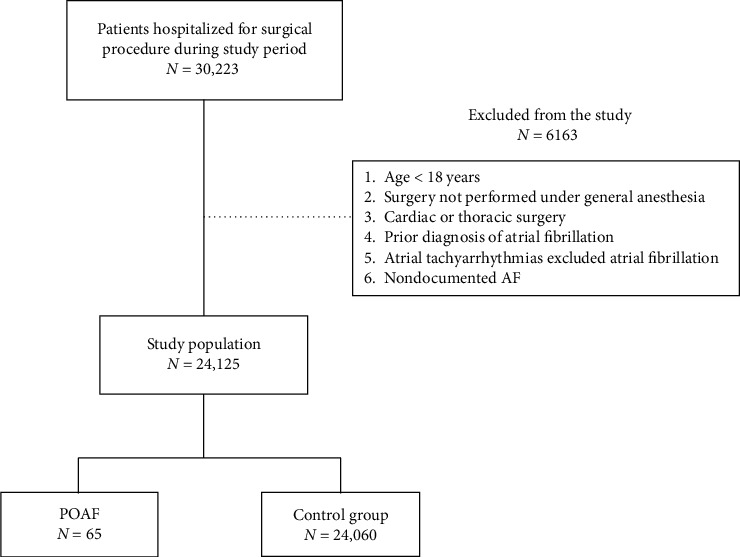
Schematic illustration of the study design and patient-selection criteria.

A total of 30,223 operations were performed at 7 surgical wards during the study period. 6163 patients were not included in the study due to failure to meet the inclusion criteria. A total of 24,125 patients included in the study were divided to two groups based on the diagnosis of AF (Figure 1).

The prevalence of POAF varies significantly, with an average rate of 2.69 per 1000 adult patients (95% CI: 2.11–3.43). Higher prevalence was observed after hip fixation and abdominal surgeries conducted in orthopedics and general surgery departments. Patients who developed POAF were older (63.8 ± 11.5 vs. 49.8 ± 18.6, *p* < 0.0001) with higher prevalence of smoking (23.1% vs. 9%, *p* < 0.0001), hypertension (69.2% vs. 23.3%, *p* < 0.0001), and diabetes (36.9% vs. 14.4%, *p* < 0.0001). Those patients had significantly higher prevalence of ischemic heart disease (30.8% vs. 7.2%, *p* < 0.0001), chronic heart failure (7.7 vs. 0.6, *p* < 0.0001), chronic renal failure (30.8% vs. 5.4, *p* < 0.0001), anemia (18.5% vs. 9.8, *p* < 0.0001), and CVA or TIA (20.0% vs. 2.4%, *p* < 0.0001) as compared to the control group (Table 2). The mean BMI was 28.89 ± 7.31, and the mean CHA_2_DS_2_-VASc was 3.78 ± 1.25.

After development of POAF, 23% converted spontaneously, and the rest were approached with medical and electrical cardioversion. Cardioversion using pharmacological agents were the most common initial approach (42.1%) as compared to rate control (37.5%) and electrical cardioversion (20.3%). Electrical cardioversion was the most efficient treatment in restoring sinus rhythm with 69.2% success rate. About two-fifth (40%) of patients with POAF were subjected to inappropriate anticoagulant use at discharge. This was mainly seen in patients who were prescribed with direct oral anticoagulants. Physicians tended to discharge patients with a lower dose of DOAC despite objective indications such as renal failure, low body weight, and advanced age. At 1 year of follow-up, we did not see a higher rate of major bleeding events nor ischemic thromboembolic events.

Among the POAF group, about two-fifths (40%) of the operation were operated electively, and the rest underwent emergency surgery. POAF development was analyzed according to the type of surgery, whether urgent or elective. There was significantly lower survival time in patients who developed POAF after emergent surgery (*p*=0.03).

There was no difference in the rates 30 days and 1 year MACE between elective and urgent POAF. Seven patients had MACE at 30 days (1 PE and 6 recurrent AF). Of these, MACE occurred in three patients who had elective surgery (11.5%) and in four patients who had an emergent surgery (10.2%). There was no statistically significant difference in survival time between patients who had elective surgery (28.9 days) and those who had emergent surgery (28.7 days; Mantel–Cox chi-square = 0.02; *p* > 0.89). This remained true after correcting for age (chi-square = 0.02, *p* > 0.90).

15 patients had 1-year MACE. Of these, MACE occurred in 4 patients who had elective surgery (15.4%) and in 11 patients who had an emergent surgery (28.2%). There was no statistically significant difference in survival time between patients who had elective surgery (312.8 days) and those who had emergent surgery (293.6 days; Mantel–Cox chi-square = 1.23; *p* > 0.27). This remained true after correcting for age (chi-square = 1.19, *p* > 0.28).

There was a significantly higher length of hospitalization in the urgent surgery POAF group (*p*=0.008). Of the patients who developed POAF, urgent surgery entailed significantly lower survival time (Mantel–Cox chi-square = 4.51, *p*=0.03).

**Figure 2:**
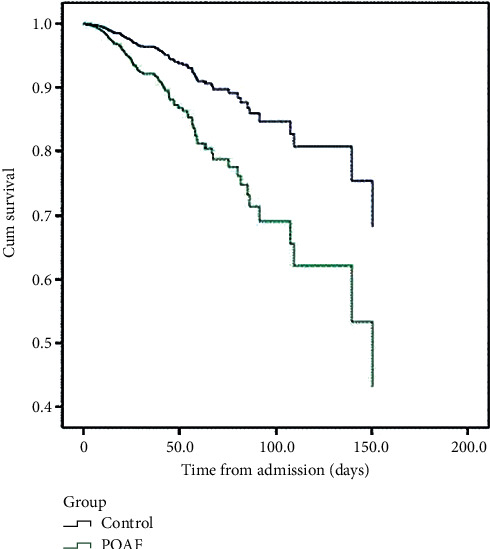
Kaplan–Mayer survival curve among study groups.

Inhospital mortality differed significantly among study groups. 182 patients in the control group died during hospitalization as compared to 13 patients in the POAF group (200 vs. 7.56 per 1000; *p*=0.001). The control group had statistically significant longer survival time than the POAF group for inhospital death (Mantel–Cox chi-square = 14.33; *p* < 0.001). After correcting for age (Cox regression model), the control group still had a statistically significant longer survival time (chi-square = 7.51; *p* < 0.006) (Figure 2).

30 days mortality significantly differed among two groups, with 239 patients in the control group died within 30 days and 7 patients in the POAF group (8.22 vs. 107.69 per 1000, *p*=0.001; Mantel-Cox chi-square = 81.26, *p* < 0.001). After correcting for age, the difference remained (chi-square = 10.62, *p* < 0.001). After correcting for smoking, hypertension, diabetes mellitus, chronic renal failure, anemia, ischemic heart disease, chronic heart failure, CVA/TIA, and CHADS_2_-VA_2_SC score, the difference remained (chi-square = 6.51, *p* < 0.01).

1-year mortality differed significantly as well. 802 patients in the control group died at 1 year as compared to 17 patients in the POAF group (261.5 vs. 3.33 per 1000, *p*=0.001). Age was not a significant predictor of 1-year mortality.

**Figure 3:**
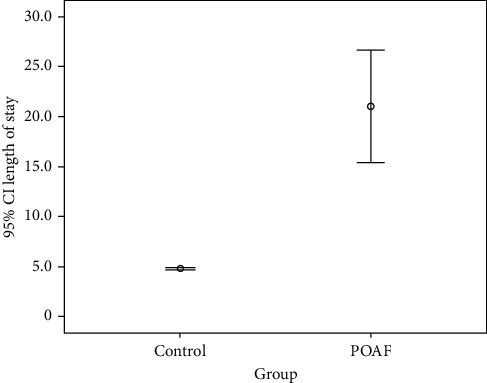
Mean length of stay among study groups.

Patients who develop POAF had a significantly longer hospital stay. LOH in the POAF group was 21.0 days (SD: 22.7; range 2–115 days; 95% CI: 15.4–26.6 days) compared to 4.8 days (SD: 7.5; range 0–188 days; 95% CI: 4.7–4.9) in the control group (Figure 3).

In patients developing POAF, 24.6% developed MACE (1.5% MI, 1.5% PE, 12.3% CHF, 13.5% AF, and 4.6% CVA/TIA).

## 4. Discussion

Postoperative atrial fibrillation in noncardiothoracic surgery is rare, as previously reported [12–20], with a prevalence of 2.69 per 1000 adult patients. The highest prevalence was observed during abdominal and orthopedic surgeries.

The low incidence of POAF may be explained by several factors such as baseline patient characteristics, shorter length of hospitalization, less cardiac trauma and manipulation, and shorter and less accurate cardiac monitoring outside intensive cardiac care.

One must bear in mind, that noncardiothoracic surgeries are usually less major, do not include extensive sternotomies, entail less blood loss, less fluid, and electrolyte imbalance, and most importantly, do not involve extensive cardiac manipulation. Moreover, many noncardiothoracic surgeries include minimally invasive techniques, whereas its usage in cardiothoracic surgeries is still limited. All the aforementioned were found to contribute to the appearance of AF [21–28].

In this study, patients who developed POAF had the same well-known predisposing factors for AF, regardless of surgeries. These patients were older with a significant higher cardiovascular risk profile, with higher prevalence of smoking, hypertension, and diabetes. In addition, these patients were more likely to have prior cardiac injury such as ischemic heart disease infarction, structural changes, and reduced left ventricle function. Therefore, we recommend surgeons to make note of this population and be wary of the potential risk.

The length of hospitalization (LOH) was significantly higher in patients who developed POAF. The mean duration was 21 days, compared to an overall mean duration of less than 5 days. This difference in LOH among groups could be explained in three possible ways, to the best of our knowledge. One is that AF constitutes a trigger that may lead to further deterioration and longer hospital stay. Second is the hypothesis that AF may be a marker of worse condition and secondary adrenergic surge. Finally, considering that LOH correlated with increased CHA_2_DS_2_-VASc scores, it is also possible that comorbidities might be the culprit for LOH, and POAF could be ominous of future prognosis.

The incidence of POAF was not found to be unexceptionally common but entailed important prognostic significance. Although rare, it must not be overlooked, and we advise surgeons to obtain cardiologic consult once it develops. Moreover, we encourage that susceptible patients with risk factors, mainly ones undergoing hip fixation or laparotomy, must be carefully examined prior to the operation and after the operation, with special care to this outcome, as it poses short- and long-term ramifications. In addition, patients should be advised to take note if any AF-related symptoms occur immediately after surgery and to inform their surgeons in such instances.

To the best of our knowledge, there are no evidence-based guidelines concerning this issue, regarding the evaluation of this phenomenon and how to address it. Once POAF has indeed developed, we recommend that, in addition to the routine care of AF, an additional cardiac monitoring should take place, preferably with a Holter monitor for another 48-hour period to examine and study the nature of the AF. Moreover, due to its long-term implications, we advise more frequent cardiac assessments, conducted by either the primary-care physician or a cardiologist, at least in the first year following surgery. Lastly, the risks of antiarrhythmic drugs are well-known [29], especially in elderly patients. We presume that, in light of the scarcity of POAF found in our study, we cannot recommend the use of preoperative prophylactic antiarrhythmic therapy. Nonetheless, if the AF is recurrent during Holter monitoring or later on, we clearly recommend it to be treated as any other AF regarding the use of antiarrhythmics.

## 5. Study Limitations

We used a retrospective methodology using data from computerized systems. Data were obtained with no ability to assess its reliability. Moreover, the follow-up to observe the incidence and development of POAF was made immediately after surgery, during the routine 36-hour postoperative monitoring alone, and therefore, the rate of POAF might be underestimated as the late development of POAF might have been underdiagnosed.

This study did not examine non-GA operations since we did not have the same rigorous follow-up monitoring as is routinely performed after GA operations, making the information less accurate. In our view, future studies investigating this phenomenon in these operations may be warranted and could possibly provide interesting, additional insights on the subject.

## 6. Conclusion

Our results indicate that POAF following noncardiothoracic surgeries is rare; however, just like postcardiac surgery, it poses a significantly poor prognostic implication. POAF is associated with a fivefold hospitalization length and a significantly higher mortality rate, both inhospital and up to one year following discharge.

## Figures and Tables

**Table 1:** Inclusion and exclusion criteria.

Criteria for inclusion	Criteria for exclusion
(1) Inpatients who underwent a surgical procedure during the study period	(1) Age <18 years
	(2) Surgery not performed using general anesthesia
	(3) Cardiac- or thoracic-related surgery
	(4) Prior diagnosis of AF
	(5) Supraventricular arrhythmia excluded AF
	(6) Nondocumented AF

**Table 2:** Baseline clinical characteristics among study groups.

Parameters	POAF (*N* = 65)	Control (*N* = 24060)	*p* value
Age (mean ± range)	63.8 ± 11.5	49.8 ± 18.6	<0.0001
	(65; 40–96)	(49; 19–104)	
Gender: male	28 (43.1)	10877 (45.2)	0.73
Smoking	15 (23.1)	2170 (9.0)	<0.0001
Hypertension	45 (69.2)	5602 (23.3)	<0.0001
Diabetes mellitus	24 (36.9)	3454 (14.4)	<0.0001
Renal failure^*χ*^	20 (30.8)	1299 (5.4)	<0.0001
Anemia^*ψ*^	12 (18.5)	2357 (9.8)	<0.0001
Ischemic heart disease	20 (30.8)	1724 (7.2)	<0.0001
Chronic heart failure	5 (7.7)	143 (0.6)	<0.0001
CVA/TIA	13 (20.0)	574 (2.4)	<0.0001
CHADS_2_-VASC_2_	3.78 ± 1.97	1.24 ± 1.33	<0.0001
	(3; 0–8)	(1; 0–9)	

CVA, cerebrovascular accident; TIA, transient ischemic attack. ^*χ*^Defined as serum creatinine level >1.5 mg/dL or estimated GFR <60 ml/L/1.73 m^2^ using MDRD. ^*ψ*^Defined as hemoglobin level <9 gram/dL.

## Data Availability

The data used to support the findings of this study are available from the corresponding author upon request.
